# Supplementary material for: Learning to Be an Orangutan—Implications of Life History for Orangutan Rehabilitation
Source: Animals (Basel). 2021 Mar 10;11(3):767. doi: 10.3390/ani11030767 (PMC8001071; doi:10.3390/ani11030767)
Supplement: Supplementary file 1 [file animals-11-00767-s001.pdf]

Preuschoft et al.

Learning to be an Orangutan – Implications of Life History for Orangutan Rehabilitation

Supplementary Material

***Table S1**: Ethogram*

**Activity budget**		
Feed	Processing, gathering and ingestion of food items, drinking	Classification:<br>• Type of food (from caretaker, forest food, non-forest food, soil, invertebrate, left-overs, water, other, unknown)<br>• Part of plant (fruit, flower, seed, stem, pith, bark, leaves, young leaves, unknown)
Rest	Sitting, lying, standing, hanging for more than 5 sec	
Travel	Movements, usually between trees and patches	
Social	Social interactions with conspecifics and/or caregivers	Classification:<br>• Social play<br>• Sexual behaviour<br>• Conflict (aggressive and submissive)<br>• Affiliation (body contact, food sharing, peering, grooming, greeting, kissing, gentle touch, hugging)<br>• Caregiving (carrying, share a nest, protection from danger, sleeping in caregiver's lap)
Other	Solitary activity, building a nest, stereotypic behaviour, other behaviour	Classification of solitary activity:<br>• Solitary locomotor play<br>• Exploration/manipulation<br>• Vigilance<br>Classification of nest building:<br>• Type of nest (ground, crotch, branch, trunk, connected trees)<br>• Quality of nest (old, rebuild, new)
**Use of Forest Stratum**		
Ground	From the ground up to a height of 1 m	
1-5 m	From >1 m up to a height of 5 m	
5-10 m	From >5 m up to a height of 10 m	
>10 m	Higher than 10 m	
**Proximity**		
Proximity 1	From body contact to 1 m distance	Classification:<br>• Human: female caregiver, male caregiver, familiar person, stranger<br>• Orangutan: name of orangutan
Proximity 2	>1 to 5 m distance	
Proximity 3	>5 to 10 m distance	
Proximity 4	>10 m distance	

***Table S2**: Intra-individual comparison of social interaction frequencies (Amalia over 3 quarters, Eska and Cantik over 6 quarters).*

		Q1	Q2	Q3	Q4	Q5	Q6
**Social human**	**Amalia**				-0.7	4.4	-3.7
	**Eska**	-1.7	9.7	2.2	-4.7	-2.6	-3.3
	**Cantik**	-6.5	-0.2	8.0	-2.3	-1.3	2.6
**Social OU**	**Amalia**				1.5	3.7	-5.1
	**Eska**	13.7	6.8	0.2	-4.3	-3.9	-13.5
	**Cantik**	15.3	2.1	4.0	2.6	-7.3	-14.4
**No social interaction**	**Amalia**				-0.1	-0.7	0.7
	**Eska**	-4.1	-3.4	-0.3	2.0	1.6	4.7
	**Cantik**	-3.3	-0.5	-2.2	-0.4	2.2	3.6

Values refer to standardized residuals (*r*). Darker colours highlight significantly higher values than expected (i.e. standardized residuals of ≥2); lighter colours highlight significantly lower values than expected (i.e. standardized residuals of ≤-2).

***Table S3**: Intra-individual comparison of stratum use (Amalia over 3 quarters, Eska and Cantik over 6 quarters).*

		Q1	Q2	Q3	Q4	Q5	Q6
**Ground**	**Amalia**				14.8	-1.7	-11.9
	**Eska**	-2.7	13.5	12.7	-0.9	-7.0	-16.4
	**Cantik**	2.2	8.0	6.7	8.0	-5.6	-16.3
**1-5 m**	**Amalia**				12.9	-0.9	-10.9
	**Eska**	3.0	2.9	2.8	0.1	-0.2	-8.9
	**Cantik**	8.3	4.5	5.3	-1.2	-4.7	-10.8
**5-10 m**	**Amalia**				0.5	1.2	-1.7
	**Eska**	-5.9	-7.6	-7.0	7.6	5.4	8.7
	**Cantik**	-3.0	-4.3	-2.4	-2.5	0.5	10.6
**>10 m**	**Amalia**				-9.9	0.2	8.9
	**Eska**	5.9	-15.4	-14.7	-5.9	5.2	25.0
	**Cantik**	-6.4	-7.5	-8.4	-4.1	8.7	14.9

Values refer to standardized residuals (*r*). Darker colours highlight significantly higher values than expected (i.e. standardized residuals of ≥2); lighter colours highlight significantly lower values than expected (i.e. standardized residuals of ≤-2).
